# Population screening for glaucoma in UK: current recommendations and future directions

**DOI:** 10.1038/s41433-021-01687-8

**Published:** 2021-08-03

**Authors:** Sana Hamid, Parul Desai, Pirro Hysi, Jennifer M. Burr, Anthony P. Khawaja

**Affiliations:** 1 NIHR Biomedical Research Centre at Moorfields Eye Hospital NHS Foundation Trust & UCL Institute of Ophthalmology, London, UK; 2 Section of Ophthalmology, School of Life Course Sciences, King’s College London, London, UK; 3 Department of Twin Research and Genetic Epidemiology, King’s College London, London, UK; 4 School of Medicine, University of St Andrews, St Andrews, Scotland UK

**Keywords:** Optic nerve diseases, Predictive markers

## Abstract

Effective population screening for glaucoma would enable earlier diagnosis and prevention of irreversible vision loss. The UK National Screening Committee (NSC) recently published a review that examined the viability, effectiveness and appropriateness of a population-based screening programme for primary open-angle glaucoma (POAG). In our article, we summarise the results of the review and discuss some future directions that may enable effective population screening for glaucoma in the future. Two key questions were addressed by the UK NSC review; is there a valid, accurate screening test for POAG, and does evidence exist that screening reduces morbidity from POAG compared with standard care. Six new studies were identified since the previous 2015 review. The review concluded that screening for glaucoma in adults is not recommended because there is no clear evidence for a sufficiently accurate screening test or for better outcomes with screening compared to current care. The next UK NSC review is due to be conducted in 2023. One challenge for POAG screening is that the relatively low disease prevalence results in too many false-positive referrals, even with an accurate test. In the future, targeted screening of a population subset with a higher prevalence of glaucoma may be effective. Recent developments in POAG polygenic risk prediction and deep learning image analysis offer potential avenues to identifying glaucoma-enriched sub-populations. Until such time, opportunistic case finding through General Ophthalmic Services remains the primary route for identification of glaucoma in the UK and greater public awareness of the service would be of benefit.

## 英国青光眼的人群筛查: 当前建议和未来方向

摘要

对青光眼进行有效的筛查有助于疾病的早期诊断和预防不可逆转的视力丧失。英国国家筛查委员会 (NSC) 最近发表了一篇综述, 研究了以人群为基础的原发性开角型青光眼 (POAG) 筛查方案的可行性、有效性和适宜性。在我们的文章中, 我们总结了这篇综述的结果, 并讨论了一些未来可能使青光眼进行有效的 (人群筛查实现的方向。英国国家安全委员会审查提出了两个关键问题: 第一, 是否有一个有效并准确的POAG筛查检测; 第二, 是否有证据表明, 筛查与标准治疗方案相比降低了POAG的发病率。自2015年这篇综述发表以来, 又有6项新的研究发表。该综述的结论是, 不建议对成人进行青光眼筛查, 因为没有明确的证据表明筛查检测足够准确, 也没有证据表明筛查比目前的治疗效果更好。英国国家安全委员会的下一次评估将于2023年进行。

POAG筛查面临的一个挑战是, 即使进行了准确的检测, 相对较低的发病率也能导致大量的假阳性转诊。在未来, 对青光眼高发人群进行针对性筛查可能是有效的。POAG多基因风险预测和深度学习图像分析的最新进展给识别丰富的青光眼分类提供了潜在的途径。在此之前, 通过普通眼科检查偶然发现的病例仍然是英国青光眼的主要发现途径, 提高公众对该项检查的认识将是有益的。

## Introduction

Glaucoma is the second most common cause of blindness globally [1]. Unlike cataract, the leading cause of blindness, glaucoma causes irreparable vision loss. This, in combination with the progressive nature of the disease, means that early detection and treatment are critical for preventing blindness from glaucoma [2]. The asymptomatic nature of mild glaucoma means that examination is required for early detection [2]. While the necessity for early diagnosis to prevent blindness may suggest that glaucoma is a good candidate for population screening, inadequate tests for the relatively low prevalence in the population have so far precluded a national screening programme. Currently in the UK, glaucoma detection is opportunistic, most frequently by optometrist assessment in the community. A recent population-based study in Northern Ireland suggests that the majority of people with glaucoma are undetected or are at least unaware of their diagnosis [3].

The UK National Screening Committee (UK NSC) regularly reviews the evidence supporting population screening to provide recommendations to the government and National Health Service (NHS) in the UK. In 2019, the UK NSC updated their 2015 review of available evidence supporting population screening for the commonest form of glaucoma, primary open-angle glaucoma (POAG) [4]. The 2019 report states that the UK NSC still cannot recommend population screening for POAG in adults due to inadequate supporting evidence. In this article, we present a summary of the findings of the 2019 UK NSC review and discuss potential future directions that may enable effective population screening of POAG.

## UK National Screening Committee External Review of Screening for Glaucoma, 2019

### Aims

The viability, effectiveness and appropriateness of a population-based screening programme for POAG were assessed by the UK National Screening Committee (UK NSC) and their review was published in December 2019 [4]. Two key questions relating to the UK NSC screening criteria were evaluated to determine whether there was any new relevant evidence published since the last review in March 2015 and therefore whether to reconsider the recommendation of the last review against population screening for POAG in the UK. These two questions considered firstly whether there is a valid, accurate screening test for POAG and secondly whether evidence exists that POAG screening reduces morbidity from the condition compared with standard diagnosis and care. The NSC commissioned the evidence review which was carried out using rapid review methodologies [5].

### Methods

A systematic search of three databases (Medline, Embase and Cochrane) was undertaken to identify relevant studies published since 1 October 2014 up to 25 March 2019 in relation to the key questions; search criteria are summarised in Table 1.

**Table 1:** Search criteria for key review questions.

Key question	Intervention	Comparator	Outcome	Study type	Exclusion criteria
What is the diagnostic accuracy of screening tests for open-angle glaucoma in the adult population?	**Tests of optic nerve structure** *Heidelberg Retinal Tomography (HRT)*, *Optical Coherence Tomography (OCT)*, *Optic Disc Photography, Retinal Nerve Fibre Layer (RNFL) Photography, Scanning Laser Polarimetry (SLP)* **Tests of optic nerve function** *Frequency Doubling Technology (FDT), Goldmann Applanation Tonometry (GAT) Non-contact Tonometry (NCT), Standard Automated Perimetry (SAP)* Any testing tools for open-angle glaucoma	Optic disc assessment and standard achromatic white on white perimetry	Measures of the predictive validity of screening tests	Studies in randomly assigned or consecutively enroled populations prioritised	Case control studies, case reports, case series, reviews, non- peer reviewed literature Non-English language publications Studies published before October 2014
Are there any RCTs assessing whether a screening programme for open-angle glaucoma in the adult is effective in reducing morbidity?	Screening programmes to identify individuals at high risk of OAG; • direct and indirect ophthalmoscopy • fundus photography or computerised imaging of the posterior pole, optic disc, or retinal nerve • pachymetry • perimetry • tonometry	Current diagnostic methods Any treatment, no treatment or placebo	Chronic OAG eye damage - measurements of visual impairment as defined by included studies	Systematic reviews, meta-analyses, randomised controlled trials	Non-English language publications Studies published before October 2014

*RCTs* randomised controlled trials, *OAG* open-angle glaucoma.

### Findings: is there a good screening test for POAG?

The review initially searched for information on the diagnostic accuracy of screening tests for POAG in the adult population to assess whether there is a simple, safe, precise, and validated screening test. When this question was assessed by the UK NSC review in 2015, one meta-analysis, one systematic review, six studies assessing functional tests and two studies assessing structural tests were analysed. Overall, the studies had small sample sizes and a wide variability in the sensitivity and specificity of the available tests were reported, deeming them unsuitable for use in population screening.

In the 2019 review, six new studies [6–11] met the inclusion criteria after full-text review (Tables 2 and 3). The studies reported POAG screening test performance results in populations with unknown ocular history; sample sizes ranged from 220 [9] to 4167 [7]. Five of the studies targeted people with a higher risk of developing POAG due to ethnicity, age, or family history [6, 8–11]. All studies used combinations of functional and structural types of screening test and employed a screening algorithm or model to determine who should be referred for a definitive eye examination (the reference standard). The results of the definitive eye examination were typically ‘no glaucoma’, ‘suspected glaucoma’ or ‘definitive glaucoma’. The studies combined the tests used at the screening examination to calculate screening performance statistics. No studies combined the same screening tests with the same cut-offs. The reported performance of individual and combined screening tests from these studies are summarised in Tables 2 and 3, respectively.

**Table 2:** Screening test performance for suspected and definitive POAG.

	Study (sample size)	Screening test	Sensitivity %	Specificity %	Positive predictive value
**Functional screening tests and intraocular pressure**					
Visual field loss	Dabasia et al. [10] (505)	FDT perimetry (≥1 missed location at p < 1% level)	62.1% (suspected POAG) 88.5% (definitive POAG)	80.5% (suspected POAG) 79.1% (definitive POAG)	NR
		MMDT perimetry (global probability of true damage ≥3.0)	51.7% (suspected POAG) 65.4% (definitive POAG)	82.8% (suspected POAG) 81.2% (definitive POAG)	NR
Intraocular pressure Cut-off points for referral ranged from >21 mmHg to >28 mmHg	Dabasia et al. [10] (505)	NCT (ORA cc) IOP > 21 mmHg	26.9% (definitive POAG) 24.1% (combined suspected/definitive POAG)	87.9% (definitive POAG) 88.6% (combined suspected/definitive POAG)	NR
	Wahl et al. [7] (4183)	NCT (at least 1 eye IOP > 21 mmHg)	44.45% (suspected POAG) 61.54% (definitive POAG)	92.68% (suspected POAG) 91.57% (definitive POAG)	17.04 (suspected POAG) 2.23 (definitive POAG)
**Structural screening tests**					
Abnormality of optic nerve and retina Cut-offs points of cup to disc ratio for referral ranged between >0.5 and >0.8	Dabasia et al. [10] (505)	OCT GCC—focal loss of volume	46.6% (combined suspected/definitive POAG) 73.1% (definitive POAG)	91.4% (combined suspected/definitive POAG) 90.3% (definitive POAG)	NR
		OCT GCC—global loss of volume	24.1% (combined suspected/definitive POAG) 46.2% (definitive POAG)	98.2% (combined suspected/definitive POAG) 97.9% (definitive POAG)	NR
		OCT RNFL thickness inferior quadrant	46.6% (combined suspected/definitive POAG) 76.9% (definitive POAG)	96.2% (combined suspected/definitive POAG) 95% (definitive POAG)	NR

*FDT* Frequency doubling technology, *NR* not reported, *MMDT* Moorfield Motion Displacement Test, *NCT* non-contact tonometry, *ORA* Ocular Response Analyser, *OCT* optical coherence tomography, *IOP* intraocular pressure, *RNFL* retinal nerve fibre layer, *GCC* ganglion cell complex.

**Table 3:** Combined screening test performance for suspected and definitive POAG.

Study	Screening test combination	Performance reported		
		Sensitivity %	Specificity %	Positive predictive value
Song et al. [9]	CDR ratio (cut-offs ranged from >0.5 to >0.65), CDR difference between eyes (≥0.2), RNFL defect and IOP ( > 21 mmHg)	NR	NR	61.4% (suspected POAG) 25.5% (definitive POAG)
Hark et al. [11]		NR	NR	78.1% (suspected/definitive POAG)
Zhao et al. [8]	Visual acuity test, CDR (>0.7) and IOP (≥23 mmHg)	97% (for some form of ocular abnormality)	92% (for some form of ocular abnormality)	NR
Boland et al. 2016 [6]	CDR (≥0.6) and FDT perimetry (2 or more missed locations at the *p* < 1% level)	66%	70%	NR
Dabasia et al. [10]	Inferior quadrant RNFL thickness and FDT perimetry	79.3% (combined suspected/definitive POAG) 100% (definitive POAG)	63.3% (combined suspected/definitive POAG) 65.2% (definitive POAG)	22.5% (combined definitive and suspect POAG) 14.8%. (definitive POAG)
Wahl et al. [7]	FDT perimetry, non-mydriatic fundus imaging and NCT	83.78 (suspected POAG) 84.62 (definitive POAG)	99.43 (suspected POAG) 99.98 (definitive POAG)	80.14 (suspected POAG) 91.67 (definitive POAG)
	NCT (1 eye IOP > 21 mmHg) or FDT perimetry (abnormal)	65.77 (suspected POAG) 100% (definitive POAG)	87.55 (suspected POAG) 86.40 (definitive POAG)	12.63 (suspected POAG) 2.25 (definitive POAG)
	NCT (at least 1 eye IOP employees aged >21 mmHg) and FDT abnormal	6.31 (suspected POAG) 15.38 (definitive POAG)	99.65 (suspected POAG) 99.54 (definitive POAG)	33.33 (suspected POAG) 9.52 (definitive POAG)

*CDR* cup-disc ratio, *FDT* frequency doubling technology, *NR* not reported, *NCT* non-contact tonometry, *IOP* intraocular pressure, *GCC* ganglion cell complex, *RNFL* retinal nerve fibre layer.

There was no agreement about the most effective combination of tests or cut-off levels that should be used in a screening examination for POAG. The screening test performance statistics reported were variable and not comparable across studies. The review concluded that there is an insufficient evidence base for a simple, safe, precise and validated screening test with known distribution of test values and agreed suitable cut-off levels.

### Findings: does screening for POAG reduce morbidity?

The review subsequently searched for studies that investigated whether a screening programme for POAG was effective in reducing the morbidity associated with the condition. The March 2015 review assessed four studies [12–15], all of which concluded that there was insufficient evidence to recommend population-based screening for POAG. Burr et al. also reported that glaucoma screening of a population selected on age is unlikely to be cost-effective and that there is uncertainty surrounding test performance as well as around engagement with a POAG screening programme [15]. The 2013 US Preventative Services Task Force recommendation statement reported concerns about overdiagnosis and possible overtreatment as not all people go on to develop visual impairment [14]. Ervin et al. did not identify any studies to provide evidence for links between whether glaucoma screening impacted on visual field loss, visual impairment, optic nerve damage, intraocular pressure or patient-reported outcomes [13].

In the 2019 review, three additional studies reporting results of screening programmes were identified [8, 11, 16]. However, none of the studies reported any data regarding treatment outcomes or overall outcomes of the whole-screening programme performance. Anton et al. performed a cross-sectional study and reported detection rates (4.1% of those screened had glaucoma or suspect glaucoma) and costs of a screening programme (1410 € per case detected) using tonometry and imaging devices in at-risk population in Spain [16]. The screening performance statistics were beyond the scope of the study and the treatment outcomes and overall screening programme performance were not reported. A study by Hark et al. examined the agreement of ocular findings between telemedicine eye screening comprising fundus photography, tonometry, and clinical information, with diagnosis made from a comprehensive eye examination in an at risk population in Philadelphia [11]. They reported 29.2% detection rate of glaucoma-related eye disease (glaucoma, glaucoma suspect and narrow angle) in those screened; the diagnosis confirmation rate was 80% after eye examination. Performance statistics were not reported. Zhao et al. focussed on the accuracy of screening tests in the development of a screening programme in an at-risk population in Baltimore, but did not report performance statistics [8].

The UK NSC review did not identify any randomised controlled trials examining the effectiveness of a POAG screening programme in reducing disease morbidity from the condition compared with usual diagnosis and care.

### Conclusion and recommendations

Following this assessment, the committee concluded that none of the existing screening protocols should be recommended for POAG in adults. This is in line with the previous UK NSC recommendation from 2015. It is also in agreement with the 2017 National Institute for Health and Care Excellence (NICE) glaucoma guideline [17] which assessed the accuracy of five risk tools to predict conversion to POAG in people with ocular hypertension; the report concluded that the current evidence on the sensitivity and specificity of risk tools for developing POAG is of moderate to low quality, with all studies having a high or very high risk of bias [17]. The next UK NSC review is due to be conducted in 2022/23.

### Limitations

Although the evidence review underpinning the UK NSC recommendation has potential minor limitations such as searching only three electronic databases and not the grey literature, the main limitation is insufficient evidence to judge whether population screening for glaucoma is worthwhile in terms of a suitable screening test and whether the benefits of a glaucoma screening programme outweigh any potential harms. Ideally, before instigating any screening programme evidence of effectiveness from a randomised controlled screening trial is required. However, conducting such as trial of glaucoma screening is unlikely to be the best use of research resources [15] until some fundamental questions regarding what any future glaucoma screening programme is trying to achieve are addressed. In particular, the target population, the type and severity of glaucoma one is trying to detect and underlying consensus definitions.

## Future directions

**Fig. 1:**
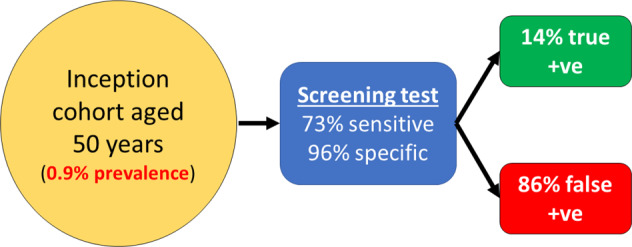
Predictive performance of a screening test (73% sensitivity and 96% specificity) when applied to an inception cohort of 50 years of age with a glaucoma prevalence of 0.9%.

One of the challenges to effective population screening for glaucoma is the relatively low disease prevalence in the general population combined with the reasonable performance of opportunistic case finding. A low disease prevalence results in a poor positive predictive value of even very accurate tests (see below). While screening for glaucoma in younger compared to older people is more likely to be beneficial in terms of reducing glaucoma disability, the prevalence of POAG in the general population selected on age alone, even in older age cohorts, is too low for a population-based screening programme to be recommended. Such a programme would overburden health services. For example, if we assume the prevalence of glaucoma in an inception cohort aged 50 years to be 0.9% [18], and the performance of the screening test or programme to be 73% sensitivity and 96% specificity [19], the positive predictive value will be relatively poor (Fig. 1). In this scenario, a positive test that will result in referral will be a false positive 86% of the time. In other words, more than 8 out of 10 referrals will be unnecessary, generating wasteful burden on secondary care services. Over-burdened secondary eye care services have been highlighted as a major problem and a cause of delays to the care of high-risk patients [20, 21]. Therefore, an efficient screening programme is required to have a higher positive predictive value (i.e., lower false positive referral rate). This may be achieved either by targeted testing of higher risk subgroups rather than inviting people selected on age alone. Modelling suggests that initiating a screening programme for a high-risk subset of a cohort aged 50 years, with an expected prevalence of POAG of around 4–5% (rather than the 0.9% in the general population aged 50 years), might be worthwhile [15, 18]. However, the systematic identification of higher glaucoma risk subgroups in the population is challenging.

One strategy could be targeting screening to families of people with POAG. First-degree relatives of glaucoma patients have been shown to have a ninefold increased risk of developing glaucoma in their lifetime compared to relatives of controls in the population-based Rotterdam Study [22]. The higher prevalence of glaucoma in first-degree relatives will improve the positive predictive value of any test, thereby reducing false-positive referrals. While a formal screening programme targeting first-degree relatives of glaucoma sufferers makes theoretical sense, the practical application may not be straightforward. For example, there would need to be a clear definition of which glaucoma patients should have their relatives screened (e.g., POAG only or other types of glaucoma as well) and these individuals would need to be accurately identified at scale nationally. Self-report of disease status is unreliable for glaucoma overall, and would likely be worse for specific sub-types of glaucoma. In the future, increasing uptake of electronic medical records may enable a digital national glaucoma registry which could inform targeted screening of first-degree relatives. However, many challenges would persist including the practicality and ethical and information governance implications of sharing health information or linkage of health records with relatives.

In recent years, there has been great progress in the discovery of the genetic determinants of POAG [23]. Over 100 common genetic variants have been identified which each contribute a small increased risk of high intraocular pressure (IOP) or POAG [24]. When combined together, these variants cumulatively can predict who will develop POAG with an area under the ROC curve of 76% [24]. Further adding genetic variants which are associated with vertical cup-disc ratio and glaucoma and creating a polygenic risk score (PRS) for POAG has also demonstrated potential for identifying individuals in a population who are at high risk for disease [25]. In the Australia and New Zealand Registry of Advanced Glaucoma (comprising 3071 advanced POAG cases and 6750 historic controls of European descent), participants in the top decile of PRS were at a 15-fold increased risk of developing advanced glaucoma compared to the bottom decile [25]. Compared to the remaining 90% of the cohort, participants in the top decile of PRS were at a 4.2-fold increased risk of advanced glaucoma.

**Fig. 2:**
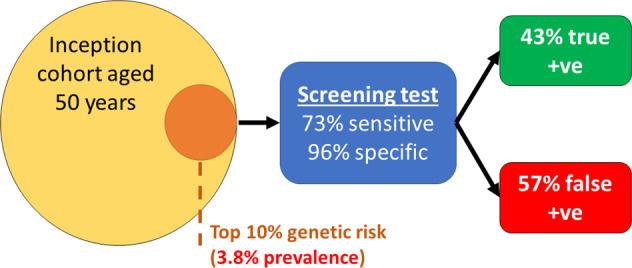
Predictive performance of a screening test (73% sensitivity and 96% specificity) targeted to a subset of the inception cohort aged 50 years with high genetic risk for glaucoma (an enriched prevalence of 0.9% × 4.2 = 3.8%).

If genetic data were available for the general population, we would be able to target a glaucoma screening programme to individuals at the highest genetic risk of developing advanced glaucoma. If we apply the 4.2-fold increased risk in the top 10% to the 50-year-old inception cohort, we would predict a glaucoma risk of around 4%. If we were then to target screening to this enriched sub-population with the aforementioned screening test (73% sensitivity and 96% specificity), the false-positive rate would fall substantially (Fig. 2). For each positive screening test result, there will be a 57% chance of a false positive. This example highlights the potential gains of targeting high-risk sub-populations, and the significant role genetic testing could play. While genetic testing is not routine in the general population currently, its affordability and applicability for multiple diseases make it a likely possibility in the future. Targeted glaucoma screening of people at high genetic risk of glaucoma is currently being examined prospectively in a large Dutch cohort [26], which if successful, could provide strong support for genotype-based targeting screening for glaucoma in the future. It will be necessary to determine the optimal testing strategy for people identified to be at high risk (e.g., top decile of genetic risk and aged 50); this may be community screening every 2 years until a glaucoma diagnosis or until an upper age-limit when it is deemed unlikely to develop vision loss due to glaucoma during remaining life. It will also be necessary to demonstrate that genetic testing is feasible and acceptable to the public, and to compare the innovative screening strategy with current case detection in a prospective randomised trial. Another major challenge is ensuring that any prediction model is generalisable to diverse populations and people of different ethnicities. To date, the majority of genome-wide association studies have examined people of European descent. While there appears to be good generalisability of genetic loci between European and Asian populations, there may be less overlap and correlation between European and African populations [27, 28]. Future work in this field must aim to improve genetic discovery in non-European ethnic groups to enable the development and validation of prediction tools that can be deployed equitably in the future.

Another potential tool to help enable effective population screening for glaucoma is artificial intelligence-assisted image interpretation. Deep learning techniques have enabled algorithms that can classify optic disc images according to glaucoma risk and the need for further examination for glaucoma [29]. In some cases, the algorithm has been trained on images graded by multiple experts, with the resultant algorithm outperforming any single glaucoma expert in independent test sets [30]. While it may be unlikely for the positive predictive value of such an algorithm to be adequate when applied to the general population, it may be that the algorithm is used to identify a subset of the population that should be screened (rather than immediate referral). Similar to Fig. 2, screening a sub-population enriched for glaucoma (as detected by an optic disc image deep learning algorithm) may be effective due to reduced false positives compared to screening the general population. In addition, multimodal algorithms incorporating data and images on visual fields, IOP and optical coherence tomography (OCT) may reach the point of adequate predictive ability, even in a general population. Ultimately, approaches that combine both genetic prediction and deep learning algorithms may be developed.

Until effective population screening or targeted screening programmes are achievable, opportunistic case finding through General Ophthalmic Services (GOS) will remain the primary route for identification of both symptomatic and asymptomatic glaucoma in the UK. The GOS (commonly referred to as the “sight test”) is provided by optometrists in community optical practice, but with distinct differences in the contract across each of the UK nations. It is provided as an NHS service for those meeting a range of eligibility criteria; criteria pertinent to glaucoma are as follows: having a diagnosis of glaucoma, aged 40 or over and either a parent, sibling or child that has been diagnosed with glaucoma, advised by an ophthalmologist as being at risk of glaucoma, aged 60 years and over [31]. The exception for this being Scotland where a sight test is provided as a universal NHS service to the whole population [32]. In addition, the contracting arrangements in all the devolved nations allow for the provision of supplementary services to improve the quality of decision-making for onward referral to specialist ophthalmic care [32–34], which include repeat measures and referral refinement recommended by NICE [17]. In England, the commissioning of these supplementary services are encouraged and will be central to the models of care for glaucoma for the Integrated Care Systems that are currently being established [35, 36]. Despite these national differences, the GOS has an established process and defined clinical and professional standards. Greater public awareness of the service would not only improve health literacy facilitating healthier choices, but also improve case finding for a range of eye conditions including glaucoma, in the population at risk.

It should be noted that the clinical and cost-effectiveness of glaucoma screening depends on multiple factors that are specific to the healthcare setting. Modelling studies in Finland [37] and China [38] have suggested that population screening for glaucoma may be cost-effective in those healthcare settings. However, it is acknowledged that such models have uncertainty and can be sensitive to the specificity of diagnostic tests and cost of screening [37]. Another factor that is difficult to predict and can influence the effectiveness of a screening programme is the attendance rate.

Glaucoma remains an important cause of avoidable sight loss in England and Wales [39]. Effective population screening would enable earlier diagnosis and prevention of irreversible vision loss. Major advances in our ability to predict glaucoma risk using genetic markers, increasingly affordable genotyping, and advances in machine learning techniques all provide promise to enable innovative solutions for effective glaucoma screening in the future.
